# Repercussions of COVID-19 Lockdown on Implementation of Children’s Rights to Education

**DOI:** 10.3390/children10030474

**Published:** 2023-02-27

**Authors:** Nurul Hidayat Ab Rahman, Mohd Zamre Mohd Zahir, Nabeel Mahdi Althabhawi

**Affiliations:** Faculty of Law, Universiti Kebangsaan Malaysia, Bangi 43650, Selangor, Malaysia

**Keywords:** COVID-19, children’s rights, lockdown, right to education, sustainable development goals

## Abstract

Lockdowns were among the control measures taken by many countries to hinder the virus from rapidly spreading. Public places had to be closed, including schools, as children are among the vulnerable groups that must always be protected. The primary purpose of this article is to explain children’s rights to education based on the Convention on the Rights of the Child (CRC) and the Sustainable Development Goals 2030 (SDG 2030). This article further investigates the consequences of COVID-19 lockdowns for ensuring that privilege for children. The article applies a qualitative method and observed that the lockdown strategy created challenges for all children with respect to receiving education, as the traditional learning pedagogy involving face-to-face meetings forcibly replaced by online learning. The principle of the best interest of the child is a primary consideration. In conclusion, governments must be responsible for providing all necessities to support education for all children, which is essential for human development.

## 1. Introduction

### 1.1. The Arrival of the Coronavirus Disease (COVID-19)

On 31 December 2019, the first 27 COVID-19 cases in humans were reported to the World Health Organization (WHO) in Wuhan, Hubei Province, China. Chinese authorities’ retroactive investigations have shown a connection between a wholesale food market in Wuhan and the severe acute respiratory disease caused by the SARS-CoV-2 virus. According to reports, the Wuhan City bazaar was the origin of this outbreak. It was scheduled to be shut down on 1 January 2020. Thirty days later, COVID-19 was declared by the WHO to be an outbreak of a Public Health Emergency of International Concern. The declaration was meant to alert countries with vulnerable health systems to prepare for the high risk of infections. The Emergency Committee believed the virus could be stopped by early discovery, quarantine, and immediate handling.

However, the worldwide crisis escalated, and in March 2020, COVID-19 was confirmed as a global pandemic. By August 2022, more than 578 million positive cases were documented globally. Even though vaccines were made available from December 2020, and 70% of the world population was fully vaccinated by May 2022 [1], to date the number of viral infection has continued to increase [2]. Consequently, several state governments used different control measures, including lockdowns, to combat the epidemic. This action has, among other things, slowed education growth and undermined both international and national development ambitions.

### 1.2. Lockdown: A Control Measure to Curb COVID-19 Virus Transmission

In the absence of proper vaccines or any antiviral medication at the earlier stage of the emergence of the COVID-19 pandemic, national responses to COVID-19 varied. The control measures can be divided into elimination and administrative rules, personal protective equipment, community protective equipment, and many others [3]. The measure known as ‘non-pharmaceutical interventions’ (NPIs) is considered the only practical option available, as it uses different techniques to reduce or delay the transmission of an epidemic virus without using pharmaceutical drug treatments. Such a mechanism aims to prevent the COVID-19 virus from spreading, as the virus can stay in the air for an extended period and easily infect people through communication, sneezing, or coughing. The NPIs implemented in numerous countries worldwide include masking, hand washing, and physical distancing, colloquially known as lockdowns orders [3]. A lockdown, also known as a curfew, quarantine, movement control, ‘shelter-in-place,’ or ‘stay-at-home,’ is generally an order made by a government to restrict societal engagement and limit movements or activities in a community. This restriction policy is usually due to health and security risks. There are two main types of lockdown, namely preventive and emergency lockdowns. The former is a preventative action plan enforced to address odd scenarios or flaws in a current system to deter or mitigate any risk or danger, and to therefore ensure the safety and security of people in that community. In comparison, the latter is implemented in a life crisis situation where a threat to lives or risk of injury to humans is imminent and sudden [3]. 

Since the first emergence of the COVID-19 pandemic, lockdowns have been initiated to protect human lives. The pandemic has resulted in an immense number of global lockdowns at the same period in history. Due to these restriction orders, people’s movements were restricted domestically, and national borders were closed [4]. China was the first country to declare a lockdown order in Wuhan and other cities in Hubei Province, which later set a precedent for similar measures. On March 9th, Italy imposed a national lockdown causing about 60 million Italian people to be placed in quarantine. Following the global trend, and because of the rising number of COVID-19 cases reported globally, the Indian government announced that the whole country would be locked down on 25 March 2020, requiring that 1.38 billion Indian people stay at home during the confinement period [5]. The number of worldwide lockdowns continued to increase, and it was reported that, on 26 March 2020, 1.7 billion people were subject to some form of lockdown. The percentage then increased to almost 90% of the world population in April 2020. The International Monetary Fund (IMF) reported that COVID-19 caused the World Great Lockdown as many countries’ economies slowed sharply, causing the deepest recession since World War II. In that critical period, many nations experienced multiple crises, including in health, education, and development, which significantly affected vulnerable groups [6].

Vulnerability is defined as the weakened capability of a person or community to predict, deal with, resist, and recover from the effects of any danger. It has two sides: outwardly, it relates to exposure to dangers, and inwardly, it relates to the capability to overcome the hazard. Vulnerability is just an individual’s risk, but it can be seen that individual risks vary within a society; and the disparities and circumstances that exist prior to the occurrence of a hazard are mostly attributable to the variations in risk [7].

## 2. Methodology

This study employed a qualitative approach to legal research. The collection of primary and secondary data included library research and the use of publicly accessible data sources, mainly the Internet. Using the content analysis approach, each piece of information was critically evaluated and analysed [8]. The article applied a qualitative method by highlighting literature from within and outside the country based on primary and secondary sources that discuss the issue of children’s rights. This qualitative methodology has its own data and has differences in quantitative data collection. These qualitative data are descriptive, using words or writing in relation to observable human behavior. This qualitative data collection has three main data sources, namely, the results of observations, trial results and written materials.

In this article, the method used is to analyse the document. Document analysis is a breakdown of the method of collecting research data from content analysis that describes the objectives and messages of whether the material is printed, published or illustrated. This article also concerns about the theoretical part which concern about the principle of the best interest of children. The best interest of the child’s principle that seeks to protect the child’s physical, psychological, and social welfare need to be uphold. For instance, Article 3(1) of the CRC reads as follows: “…in all actions concerning children, whether undertaken by public or private social welfare institutions, courts of law, administrative authorities or legislative bodies, the best interests of the child shall be a primary consideration”. According to Mary George and Noor Aziah Mohd Awal, the rights embedded within the CRC itself provide a cohesive structural framework to Article 3(1) and guide the way towards a result that is thought to be in the child’s best interest. Children’s rights in Malaysia have developed since Malaysia acceded to the Convention on the Rights of the Child (CRC) in 1995 and introduced the Child Act in 2001. The best interests of the child are stated in Malaysian legislation like the Child Act 2001. For instance, in Section 30 (5), Child Act 2001 (Act No. 611, Malaysia). Whereas, Section 30 (1), Child Act 2001 (Act No. 611, Malaysia) with regard to if a Court for Children is satisfied that any child brought before it under Section 19 or 25 Child Act 2001 (Act No. 611, Malaysia) is a child in need of care and protection. In order to determine what is in the child’s best interests, each issue must be viewed from the child’s point of view, with a view towards considering his or her opinions and with the goal of ensuring that the child’s rights are respected (Law Insider, 2023). Any decision involving a child should therefore be informed by what is, given the child’s age and maturity, objectively best for that child. Therefore, in this article, the principle of the best interest of children will be seen in the context of education.

## 3. Findings and Discussion

### 3.1. Inefficiency of Children’s Rights to Education Due to COVID-19 Lockdown

The principle of the best interest of the child is a primary consideration. The ‘best interest’ principle is prevalent in medical law, family law and is used in the United Nations Convention on the Rights of the Child (CRC) in relation to the child [9]. In general, every individual has the right to health and to decide what he wants to do with his body [10]. Health-related problems are an important issue [11]. Every individual has the right to medical treatment and health care [12]. Health problems are also very important in the life of a society [13]. Therefore, it is very important to protect the health of individuals. 

To halt the COVID-19 virus transmission, many countries were forced to resort to lockdowns and the closure of national borders. At the same time, the infected person, travelers, and children are put under quarantine. Nonetheless, prolonged lockdowns or quarantines caused people to lose their sources of income. Massive economic shutdowns for an extended period had impacted many industries to succumb, and businesses had to suspend their operation or go through downsizing, causing employees’ service termination [14]. Other than that, many had to face salary deductions or delayed payments. Such a situation negatively impacts the family’s finances, whereby people can no longer maintain their lifestyle, and some barely make it for daily survival. Sustaining human rights is exceptionally challenging during the world economic recession. With the family financial breakdown and prolonged lockdown period, the protection of children’s fundamental rights will be severely affected, including the right to education [7]. In addition, social issues such as extreme poverty, the digital divide, hunger and malnutrition, suicide, and other health problems accelerated during the pandemic also obstructed children’s education rights to be implemented [7].

When discussing about the best interest of the child, we have to look to his or her fundamental rights first. Article 2 of the CRC states every child is entitled to all fundamental rights provided under the Convention. The core principle embedded in the article is equality, whereby children used to be discriminated against due to their lack of physical, mental, and emotional capabilities. For example, vulnerable children groups have to face more risk of being left out in digital education world because of socio-economic and geographical factors. They do not have access to online learning system because lack of modern devices such as computer, smart phone or the internet. Other than that, reference can be made to Article 6, which explains the relationship between the right to survive and develop as part of living. The right to development is linked to rights to education mentioned under Articles 28 and 29. It also supports the right to access information and participates in the decision-making process stipulated under Articles 12, 13, and 17. 

Nevertheless, due to COVID-19, the governments of a number of states have proclaimed mobility limitations, public health measures, governance, socioeconomic measures, social distance, and lockdown. Such reactions have had very substantial social, economic, and environmental effects. For instance, the virus slowed down the world’s financial activity, resulting in a worldwide economic strike that subsequently exacerbated severe poverty among global inhabitants [15]. Most importantly, children’s education and development rights are neglected besides health and malnutrition problems. Three factors are necessary for good child development, mentioned by Chan are steady, responsive, and caring caregiving with learning opportunities; safe, supporting physical settings; and sufficient nutrition [16]

If the ineffective implementation of education rights continues, children will be forced to live a low level of life and prevented from achieving success [7]. The greatest impact of this vicious cycle is the formation of an uneducated, ignorant generation that will drift into a deep destitution hole. The children will have to live in hardship as they are not given a proper platform to maximize their life potential and spread their wings of success. Without fair chances, their life development rights are violated, and the cruciality of the situation will occur when the world’s sustainability seems far from reality. Education is recognized as one of the human basic rights as well as the means for the realization of other fundamental rights [7]. It can either contribute to enhancing the quality of other rights if it is well protected or jeopardize them if violated. Therefore, this article aims to elaborate on children’s rights to education and analyze the effects and challenges of COVID-19’s lockdown on the implementation of education rights.

### 3.2. Education: A Legal Right for all Children

Education is vital in shaping children’s personalities and developing their talents and abilities. Through education, children are taught to understand their basic rights and the importance of respecting other people’s rights, cultures, and differences. Thus, States need to guarantee the execution of such rights to protect the environment and help young people live peacefully. Throughout the human history, right to education has received many recognitions such as Universal Declaration on Human Rights (UDHR), and International Covenant on Social Economic and Cultural Rights (CESR). The importance of such right also been adapted by other treaties such as the Convention on the Elimination of Racial Discrimination 1965 (CERD), Convention on the Elimination of Discrimination Against Women 1979 (CEDAW), Convention on the Rights of Migrant Workers 1990 (CRMW) and the Convention on the Rights of Persons with Disabilities 2006 (CRPD). The significance of education’s right also is mentioned in many international documents such as resolution, policy, guidelines, strategy or plan and many others. 

For example, according to the Declaration on the Right to Development right to education refers right to participate in, contribute to, and enjoy economic, social, cultural, and political development, in which all human rights and fundamental freedoms can be fully realized. In order to materialize development rights, an individual must be occupied with information, knowledge, and skills. Based on Article 8 of the Declaration, states are responsible for ensuring that all necessary measures are taken at the national level to materialize the right to development. Hence, the opportunity to access basic resources, education, health services, food, housing, employment, and income distribution must be given equally to all without judging a person’s life background.

Nevertheless, the most comprehensive articulation of education rights exclusively for children is specified under the CRC. Article 28 of the CRC recognizes children’s rights to education, whereby primary education must be made compulsory and available free to all. At the same time, secondary schooling must be encouraged by fully sponsored education or financial assistance. Furthermore, tertiary education or vocational skills must be accessible to all children. As constructed by Article 29, learning is essential to develop children’s personalities and talents or enhance their mental and physical abilities. Besides that, it is also necessary as it helps to build respect for human rights, the child’s parents, cultural identity, language, national values, and the natural environment. Moreover, education will shape the child to nurture good characteristics such as being responsible, understanding, peaceful, tolerant, just, and kind to everyone, including persons of indigenous origin. Indigenous people refer to unique social and cultural group with a common ancestry to the land and resources on which they now reside, were occupied, or were displaced. 

Furthermore, protection of the right to life is mentioned under Article 6 and is linked to survival and development rights. The State Parties must also guarantee the development rights of disabled children based on Article 23, which safeguards their freedom to receive education or training to prepare and create opportunities for them to get involved in the industry. Participation right is an extended right of education. It allows the children to give their opinions on issues affecting their life freely. Based on Article 12, adults must respect children’s views and wishes by considering matters tabled by children. Article 13 continues by highlighting children’s freedom in sharing their understanding of knowledge, feelings, and thoughts. They can express them by speech or using any written form as long as it does not harm anyone.

Nonetheless, in order to be able to do that, children must be well-equipped with sufficient knowledge and skills. It is to ensure that they are able to deliver their ideas well and that the inputs obtained can be used to benefit their life. Thus, Article 17 protects children’s right to access information from various sources like the internet, radio, television, newspapers, books, and other sources. To guarantee that the input can is well delivered to children, the media need to share every information in different languages. However, adults must monitor and filter the overflowing inputs to children in today’s borderless world to avoid any risk or harm. The importance of the right to education for children’s development is highlighted in Article 32, whereby child labor or economic exploitation must be prevented to safeguard their future. Through education, children’s life status can be enhanced, and their safety becomes more assured. That condition will contribute to the fulfillment of other CRC provisions such as protection from violence (Article 19), social and economic help (Article 26), protection from harmful drugs (Article 33), protection from sexual abuse (Article 34), prevention of sale and trafficking (Article 35) and many others. 

The recognition of education’s rights is also crafted beautifully under the SDG 2030 [17]. Besides Goal 4, which is dedicated to education rights, several other goals touch on the same privilege. Goal 4 main objective is to ensure inclusive and equitable quality education. It also aims to promote lifelong learning opportunities for all [17]. Aside from 10 targets, SDG 4 focuses on free, equitable, and quality education for all children from early childhood development until secondary education. Access to technical, vocational, and tertiary education, including university, must be guaranteed to support them with employability skills to secure decent jobs and continue with a sustainability plan [17]. Other than that, Target 3.7 explains sexual and reproductive education while Target 13.3 discusses climate change education [17]. Based on the preamble of the 2030 World Agenda, item 7 mentioned the importance of guaranteeing access to quality education at all levels, other than health care and social protection [17]. Item 15 reported that access to education has greatly improved over past generations and encouraged technological development or innovation, global interconnectedness, and other related matters which able to aid human progress through education and information access [17]. Item 20 and 25 raised non-discriminatory issues on access to education. Other than that, item 26 acknowledged the importance of education in healthcare, while item 37 relates education with the sport [17]. In short, the essence of human growth is not static and has altered throughout time. Human development generally relates to economic progress in its early stages, which is continually focused on national revenue, but later defined as progressive social, political, and economic developments. The goal of development is to help people improve their abilities so they can have a good standard of living and give them more choices, which in this context refers to the significance of education.

The Table 1 below shows summaries of the recognition and importance of children’s rights to education based on the CRC [18] and SDG 2030 [19].

Based on these key international documents, education rights for children are recognized for many purposes, for instance, health, safety, security, socioeconomic and many others. The right to education is intertwined with several other fundamental freedoms, including the rights to knowledge, speech, participation, assembly, and association. These rights are vital for children’s education, which equips them with the information and skills necessary for subsequent development [7]. The provisions listed proved the significance of education as a foundation to support children’s growth in all aspects of life. As development is the ultimate goal of protecting their fundamental rights, the states must establish a solid administration system that can secure maximum equal rights to education at all times. For example, in high-stakes situations such as natural disasters, disease outbreaks, riots, or war. Besides health, education for children must become countries’ utmost priority.

Nevertheless, humans encountered an unprecedented global challenge to sustain the enforcement of human rights when the COVID-19 pandemic hit the world in 2020. It has led to a dramatic loss in people’s livelihoods, safety and health, employment, food systems, and many others. Regarding education coordination, modern pedagogy that fully utilized technological devices and the internet was forced to be administered worldwide due to COVID-19 lockdown. Even though the teachers and students are not fully prepared for virtual education arrangements, they are not given many options but to adapt swiftly to unforeseen circumstances. Besides lack of basic knowledge and skills, scarcity of devices and an internet connection are among the daunting challenges of the e-learning system. These crucial consequences of COVID-19 lockdown order on children’s education rights should be identified and analyzed to understand and deal with the problem immediately. Through the essential lessons, countries must spare no effort in accomplishing education rights for all children during this pivotal period [7].

### 3.3. Repercussions of COVID-19 Lockdown on Right to Education for Children 

The COVID-19 pandemic has disrupted many industries as lockdown measures had been ordered worldwide. The crisis also had significantly affect the education system across all regions whereby thousands of learning institutions had been ordered to closed [7]. Some countries had to shut the school for months, and some had exceeded more than a year of closure. Based on the United Nations Educational, Scientific and Cultural Organization (UNESCO) up until October 2021, Uganda had been recorded as the country with the most extended school closure, which lasted for 83 weeks, followed by Bolivia, India and Nepal at 82 weeks. Honduras and Panama both recorded 81 weeks of school closure. More than 635 million students worldwide are affected by their governments’ complete or partial school closure orders. 

During the COVID-19 shutdown, various educational institutes, notably Malaysia, were compelled to close. After nearly two years of nationwide school closures, which started on March 18, 2020, the nation is facing negative effects such as student attrition and academic stagnation. According to the Ministry of Education, between July 2021 and March 2022, 21,316 students, or 0.22%, dropped out of school. According to UNICEF and UNFPA, approximately 65% of parents cannot provide weekly allowance for their children, while 46% have trouble providing face masks. Up to 30 percent of parents are unable to afford to send their kids to school during a pandemic due to the resulting financial problems. During the pandemic, classes were suspended, especially for children from low-income families living in rural areas [20]. In addition, there was no longer any access to testing and evaluation methodologies on a regular basis. As a result, several nations made the decision to postpone the final exams, including Kuwait. In comparison to the years before the epidemic, the high school graduation rates in all three school divisions of Kuwait saw a significant improvement as a direct consequence of this choice. In addition to this, there was a large change upwards in the distributions of the cumulative percentage. This effect, in turn, caused worries over post-high school admissions related to potential academic failure in the future [21].

Maintaining safe school operations or resuming schools after a shutdown requires several factors. Likewise, the professional development of educators in all sorts of educational institutions must be reconsidered. All educators around the world are required to use blended learning teaching approaches for both teaching and learning. There are numerous digital educational supplies, including the world’s largest Massive Open Online Courses (MOOCs), an integrated platform of free online courses that cover subjects from high school onwards to higher education, as well as skill-based courses, to ensure that every student has access to ICT-based learning materials. In addition, there are several more virtual learning platforms, including Coursera, Udemy, Edx, Udacity, and others. In addition, several prestigious worldwide institutions offer upskilling webinars, short-term courses, etc., in order to educate and equip instructors with e-learning tools and technology. As a strategic plan to satisfy their professional needs in the post-COVID-19 pandemic era, the new teaching-learning methods have shifted towards a blended learning approach that must be managed from all six components, namely learning flexibility, online learning, study management, technology, classroom learning, and online interaction [22]. 

Despite of that, the education sector endured as it underwent tremendous evolution, bringing teaching and learning pedagogy to the more sophisticated, digitalized world. Nevertheless, the prerequisite of digital education is a computer system that can be divided into hardware and software. Hardware means the physical and visible components like monitor, central processing unit known as ‘CPU,’ keyboard, and mouse. Software is the collection of instructions, procedures, routines, and documentation used to control and execute different functions on a computer system. Two significant types of software are system and application; the former work independently, and the latter depend on it. System software is the base where the programs run in the setting, whereas application software’s role is to interact with users. These hardware and software requirements for online learning settings are usually expensive and beyond reach for vulnerable children [14]. 

Dropout rates may be reduced with the use of online education. Thus, the new approach is being used, even though some pupils were excluded due to a lack of technological devices and internet access. As a consequence of the epidemic, many families have lost their jobs and hence their ability to provide their children with suitable educational materials. To begin with, it’s expensive to use the technology that is required, such as computers, tablets, mobile phones, and others. In addition, some of the kids in the household could have to miss school if they don’t have enough electronics for everyone. Prioritizing basic needs is a must during a family recession. UNICEF Malaysia and UNFPA data show that smartphones are far and away the preferred method of accessing the internet and online education among Malaysian children. E-learning requires not just access to technology but also internet and online applications. Internet connectivity is not always readily accessible, especially in less populated or rural places. Poor or unstable internet access is another obstacle to youth e-learning. In addition, there are certain programmes that you’ll need to buy, and they might be rather pricey. Up to 30 percent of children in Malaysia’s low-income families did not have full access to the internet or mobile learning resources throughout the outbreak. Teo Nie Ching, a Malaysian member of parliament, claims that 37 percent of pupils in the Ministry of Education in Malaysia do not have access to computers during the current COVID-19 pandemic. That’s the equivalent of 1.7 million "digital poor" kids in elementary and secondary school [23].

The concept of vulnerability is always associated with risk, whether because of personal or environmental factors. However, the idea had broadened whereby vulnerability can include social, health, demography, culture, and many other factors. The ‘term’ vulnerability in a human rights context denotes the state encountered by disadvantaged populaces. For example, people faced marginalization, banishment, discriminatory conduct, or mistreatment. These circumstances lead the vulnerable groups to get exceptional care or attention and even protection from a legal point of view to sustain their survival. Groups considered ‘vulnerable’ differ from one field to another, depending on the study’s objective. According to Winkler, marginalized communities are often the poorest. Social marginalisation and economic inequality are strongly linked. Even human rights arguments have converged on economic disparity based on income or wealth [24]. Among others, children, ethnic minorities, people with disabilities, the poor, and the elderly are common vulnerable groups. Children are always considered vulnerable due to physical ability, mental maturity, and emotional stability. The Convention on the Rights of the Child 1989 (CRC) supports the statement that children’s protection starts before birth. Children’s development state is fragile compared to adults; hence they own a special place within human rights protection. The situation becomes more complex if it involves vulnerable children such as children who:-(i) do not have parental care or are at risk of losing a parent; (ii) living on the streets; (iii) smuggled; (iv) living in various conflicts, natural disasters, or wars; (v) outbreaks of disease or disability; poor and others [25]. 

During COVID-19 lockdown phases, those disabled, sick, poor, suburban, and rural children stretch to survive by relying on minimal basic needs like shelter, clothes, and food. Many volunteer groups that focus on rights to education are forced to change direction by prioritizing cases related to COVID-19. These organizations’ budget allocations or human resources have to be divided or fully granted to aid various COVID-19 emergency crises instead of concentrating on education issues. Therefore, children living in poverty find that COVID-19 lockdown creates even a harder situation for them to claim and exercise their education rights. It is rather hard for them to acquire rudimentary skills such as reading, writing, and counting by attending traditional school sessions, not to mention accomplishing school via online teaching. Even though they are fortunate enough to acquire access to an online system, they will have trouble operating the web-based, mobile, or computer-based learning system as they necessitate a whole set of new proficiency and expertise. One needs to be computer and internet literate to communicate with the online population and contents. Other than that, the internet connection is lagging or non-available in remote or undeveloped areas. Such a situation causes these vulnerable children to expect less change in their life prospects and it demotivates them from finishing schooling. Besides relying on limited government and community assistances, these unfortunate children usually will end up stopping schooling to focus on life survival mechanisms. 

Television broadcasts with instructional material to extend students’ learning were another popular learning arrangement in many nations. In other countries, such as Greece, Korea, and Portugal, TV programmes primarily targeted to younger students in elementary school, who may have had trouble accessing online learning platforms or performing self-directed study. TV broadcasts are another option to reach students who do not have access to online education. Despite these benefits, broadcasts may be confined to just a few themes owing to the limited time allotted to these TV programmes. In Spain, for example, two stations covered one of five courses every day, which are Spanish, mathematics, social science, natural sciences, arts and physical education.

In short, the execution of online learning during the COVID-19 lockdown is advantageous for some of people as it stops the virus from spreading or infecting more people. It also reduces the costs of program fees, commuting expenses, and additional budget for curriculum activities and food. E-learning program enables the students to access the materials anytime and anywhere, which helps to save the digital user’s energy and time. Other than that, the flexibility offered by the new pedagogy allowed the teachers and students to experience a diversity of learning methods and techniques which is more interactive. It encourages students to solve a problem independently, think creatively and maintain their interests or focus. 

Nevertheless, the discrepancy in the acceptance of this online learning system during the pandemic epoch demonstrates a large gap from a socio-economic point of view, known as the ‘digital divide.’ The Organisation for Economic Co-operation and Development (OECD) defines the term as the disparity between individuals, households, businesses, and geographic areas with different socio-economic levels in terms of: (i) their access to information and communication technologies (ICTs); and (ii) their use of the internet for a variety of activities. Since the COVID-19 epidemic reaches its peak, the digital and socioeconomic divide will become more pronounced and tumultuous, as the majority of learning strategies are undertaken online. The responsible parties must then consider all of these consequences to ensure equal educational opportunities are given to all children to maximize their full life potential.

## 4. Recommendations

The sudden pandemic attack has drastically changed the entire education system globally. However, considering the importance of education has caused the world to adapt to the online education system immediately. Through this new system, education can still be delivered to students through modern approaches that are simpler and more advanced. The pedagogy has many benefits regarding efficient methods, safety, and saving energy and time. Even so, not all community members are ready to use this modern method because its implementation requires several pre-conditions, such as gadgets and the internet. Without fulfilling these conditions, virtual learning cannot be materialized, and children will lose the opportunity to learn and more importantly, their basic human rights, namely rights to education, information, participation and development, are violated. 

Thus, all countries must develop a comprehensive development plan to tackle the digital divide issue related to socio-economic and geographical factors. The responsible government must establish a solid exit strategy if the traditional teaching method is unable to be practised. The government must ensure affordable devices and good-bandwidth internet services are available to everyone to meet everyone’s needs. The affordability capacity must be increased to avoid barriers or discrimination in society. Besides offering gadgets or internet services at affordable prices or subsidies tariffs, the government can also offer financing to encourage children from lower-income families to afford the essential tools. Most importantly, the internet infrastructure must be developed in all parts of the world where humans live. It ensures that valuable knowledge and information can be relayed to every child. The internet broadband’s inception works much faster than traditional dial-up connections, which are still used in suburban or rural areas. The problem arises because most telecommunication businesses find that these locations cannot provide much profit from a financial point of view. The profit factor has caused many residents in the inland areas to be marginalized and neglected in terms of consistently receiving the latest and most effective information. It caused them to be incompetent in participating in decision-making even though the central issues discussed are highly related to the children’s lives. Youngsters’ thoughts are usually ignored because they are deemed immature or incapable of giving good ideas. Hence, the core infrastructure for online education must be developed without rigidly calculating the profit margin.

Other than that, users, including children, must be empowered with relevant knowledge and skills. The public, specifically children, must be digitally literate to utilize the online applications or contents fully. A new education plan or syllabus to support the e-learning system must be developed to prepare the future generations with the comprehension and capability to use technological devices and related technology efficiently. Teaching staff must also be given an intensive course to ensure they can handle online classes.

The administrating governments must always adhere to the legal obligations stipulated under the treaty, namely the CRC, and guided by principles stated under other international documents such as the Declaration on the Right to Development and the 2030 World Agenda. The 196 State Parties to the CRC must work diligently to fulfill the responsibilities toward their countries’ future generations. The best interest of the child’s principle that seeks to protect the kid’s physical, psychological, and social welfare need to be uphold at all time. Through the principle, it requires public or private authorities to review whether this condition is met whenever a decision must be made on behalf of a child, and it ensures that his long-term interests will be considered. It must be employed as a unit of measurement when opposing interests exist. Other than that, violating international law may subject the country to diplomatic pressure or face economic sanctions, and it will tarnish the country’s reputation globally. Moreover, undedicated attitudes and ineffective measures taken by countries in fulfilling their responsibilities toward children will disrupt the foundation of the global agenda or target, such as the world sustainability plan. Thus, all countries must precisely understand their duties and conform to them. For example, countries recognizing children’s participation rights mentioned in Article 12 of the CRC and elevating their status as agents of change as specify by SDG 2030 must continuously secure their rights to education. The gap in digital access that existed because of socio-economic reasons must be securely closed to bridge all people as one community. The online learning system, which comes alive due to the lockdown measures taken by worldwide countries, must guarantee that the children’s rights to education are upheld, and their interests are protected at any time.

## 5. Conclusions

In conclusion, the goal of this research study was to conduct an analysis on international documents that protect the rights of children with respect to attaining education. After understanding countries’ legal obligations in providing the privilege to the younger generation, this article further investigated the impacts of the COVID-19 lockdown in realizing the education rights of children. The lockdown measures taken by many countries worldwide changed the education system, whereby it is revived via the modern digital world. The e-learning system, however, has rendered challenges to all, especially vulnerable children. The digital divide crisis must be addressed as many children still lack technological devices, basic skills and Internet services for accessing virtual learning programs. Henceforth, the government needs to fill the gap in order to ensure education rights for children and safeguard their best interest and other rights related to them.

## Figures and Tables

**Table 1:** Summary on the recognition and importance of children’s rights to education based on the CRC [18] and SDG 2030 [19].

Instrument	Article/Goal	Content
**Convention on the Rights of the Child**	**Article 28** **Access to Education**	1. States Parties acknowledge that children have a right to receive an education, and to realise this right in a manner that is both progressive and founded on the principle of equal opportunity, they should, in particular: (a) Make primary education mandatory and make it available for free to everyone; (b) Encourage the development of different forms of secondary education, including general and vocational education, make them available and accessible to every child, and take appropriate measures such as the introduction of free education and offering financial assistance in the event that it is required; (c) Ensure that everyone has the opportunity to pursue higher education, regardless of their financial situation, using whatever methods are available; (d) Ensure that all children have access to educational and vocational information and guidance; (e) Take steps to increase students’ likelihood of attending school on a consistent basis and lower the number of students who drop out of school. 2. The State Parties are obligated to take all steps necessary to ensure that the administration of school punishment is carried out in a manner that is respectful of the child’s inherent worth as a human being and is in accordance with this Convention. 3. The States Parties shall promote and encourage global cooperation in matters regarding education, in specific with the aim of contributing to the elimination of ignorance and illiteracy throughout the world and facilitating access to scientific and technical knowledge as well as modern teaching methods. 4. The States Parties shall ensure that the provisions of paragraphs 1 and 2 are implemented. In this respect, special attention must be paid to the requirements of developing countries
	**Article 29** **Aim of Education**	1. All of the parties have agreed that the child’s education should be focused on the following: (a) The development of the child’s personality, abilities, physical and mental competences to their full potential; (b) the development of respect for fundamental liberties and human rights, as well as the emphasis in the United Nations Charter; (c) the development of relations with the child’s parents, his or her own cultural identity, language, and principles, as well as national values of the country in which the child lives and the country from which the child comes; 2. No provision of this article or of article 28 should be construed in such a way as to infringe on the freedom of individuals or bodies to establish and direct educational institutions, subject always to the observance of the principle set out in paragraph 1 of this article and the requirement that the learning provided in such institutions conform to such basic standards as the State may lay down and establish.
	**Article 6** **Life, Survival and Development**	1. The States Parties acknowledge that the fundamental right to life belongs to each and every child. 2. The Parties must make every effort to guarantee the child’s continued life and healthy growth to the greatest degree practicable.
	**Article 23** **Children with Disabilities**	3. In acknowledgement of the unique requirements of a child with a disability, the assistance provided in pursuance of paragraph 2 of the present article shall be made available without charge, whenever this is feasible, and shall be tailored to ensure that the child with a disability has effective access to and receives education, training, health care services, rehabilitation services, preparation for employment, and recreation. This provision will take into account the monetary capacity of the child’s parents or others who are responsible for the child’s welfare.
	**Article 12** **Respect for Children’s Views**	1. The States Parties shall ensure that every child who is able to develop his or her own opinions has the liberty to express those opinions in all aspects that concern the child, with the opinions of the child being given due weight in accordance with the child’s age and maturity level. This right applies only to children who are old enough to form their own opinions. 2. To accomplish this goal, the child shall in particular be given the opportunity to be heard in any judicial or administrative proceedings that affect the child, either directly or through a representative or an appropriate body, in a manner that is consistent with the procedural rules of the national law. This chance shall be afforded to the child in a way that is in line with the procedural rules of the national law.
	**Article 13** **Sharing Thoughts Freely**	1. The child shall have the right to freedom of expression; this right shall include the liberty to seek, receive, and impart information and ideas of any kind, regardless of frontiers, orally, in writing or print, in the form of art, or via any other mainstream press of the child’s choice. 2. The child will have the right to an education. Part of this right is the freedom to look for, get, and share ideas and information of any kind, regardless of borders. 3. The application of this right may be limited by specific restrictions; nevertheless, these constraints shall only be such as are stipulated by law and are crucial: (a) For the respect of the rights or reputations of others; or (b) For the protection of national security, public order, public health, or public morals.
	**Article 17** **Access to Information**	The State Parties recognise the important role that the mass media play and agree to make sure that children have access to information and materials from a variety of international and national sources, especially those that try to improve the child’s social, spiritual, and ethical well-being as well as their physical and mental well-being. Inspire international collaboration in the production, exchange, and dissemination of such material and data from a broad range of cultural, national, and international sources. (a) Encourage the mass media to disseminate information and material of social and cultural benefit to the child in accordance with the spirit of Article 29. (b) Encourage international co-operation in the production, exchange, and dissemination of such data and information from a range of cultural, local, and foreign sources; (c) Promote the creation and distribution of children’s books; (d) Inspire the mainstream media to pay special attention to the linguistic requirements of children who are members of underrepresented groups or indigenous people, and motivate them to do so. (e) Encourage the creation of appropriate rules to protect the child from information and material that is bad for his or her health, keeping in mind the requirements of paragraphs 13 and 18.
**Sustainable Development Goals 2030**	**Goal 4** **Quality Education**	4.1 Ensure that by the year 2030, all girls and boys have had the opportunity to finish a primary and secondary education that is free, equitable, and of high quality, resulting to learning outcomes that are relevant to Goal-4. 4.2 By the year 2030, make it a priority to guarantee that all girls and boys have access to high-quality early childhood care, and preprimary education in order to prepare them for elementary school. 4.3 By the year 2030, guarantee that women and men have the same access, on an equitable basis, to affordable and high-quality postsecondary, technical, and vocational programs, inclusive university. 4.4 Increase by a significant margin, by the year 2030, the number of young people and adults who possess the required skills, including the technical and vocational skills necessary for employment and respectable occupations, as well as for entrepreneurial endeavours. 4.5 Aim to eradicate gender gaps in education by the year 2030 and guarantee equitable access to all levels of education and vocational training for those who are disadvantaged, including individuals with disabilities, indigenous peoples, and children living in precarious conditions. 4.6 By the year 2030, make it a priority to ensuring that all children and a sizeable majority of adults, including both men and women, are literate and numerate. 4.7 Ensure that all learners have acquired the skills and competencies necessary to achieve sustainable growth by the year 2030. This can be accomplished in a variety of ways, including education for sustainable development and sustainable lifestyles, human rights, gender equality, promotion of a culture of peace and non-violence, promotion of global citizenship, and awareness of cultural diversity and of culture’s contribution to sustainable development. 4.A Construct and improve educational facilities that are considerate of children, people with disabilities, and gender, and that provide secure, nonthreatening, inclusive, and productive learning environments for everyone. 4.B By the year 2020, there should be a significant increase in the number of scholarships that are made available to developing countries, in particular the least developed countries, small island developing States, and African countries, for the purpose of enrolling in higher education, including vocational training and information and communications technology, technical, engineering, and scientific initiatives, in developed countries and other developing countries. 4.C By the year 2030, the supply of trained teachers should be significantly increased. This should be accomplished in part via international collaboration for the education of educators in developing nations, particularly the least developed countries and small island developing states.
	**Target 3.7** **Education on Sexual and Reproductive Health**	By the year 2030, guarantee that everyone has access to sexual and reproductive health care services, particularly those pertaining to modern contraceptives, education and awareness as well as the incorporation of reproductive rights into national plans and initiatives.
	**Target 13.3** **Climate Change Education**	Efforts should be made to support education, awareness-raising, and the institutional and human ability to combat climate change, respond to its effects, reduce their severity, and provide early warnings.

## Data Availability

No new data were created or analyzed in this study. Data sharing is not applicable to this article.
